# Deceptive Measures of “Success” in Early Cancer Detection

**DOI:** 10.3390/curroncol31090380

**Published:** 2024-08-30

**Authors:** Nicola Cirillo

**Affiliations:** 1 Melbourne Dental School, Faculty of Medicine, Dentistry and Health Sciences, The University of Melbourne, Carlton, VIC 3053, Australia; nicola.cirillo@unimelb.edu.au; 2 School of Dentistry, University of Jordan, Amman 11733, Jordan

**Keywords:** early diagnosis, cancer, stage distribution, survival, overdiagnosis

## Abstract

Early detection of cancer is considered a cornerstone of preventive medicine and is widely perceived as the gateway to reducing cancer deaths. Based on this assumption, large trials are currently underway to evaluate the accuracy of early detection tests. It is imperative, therefore, to set meaningful “success criteria” in early detection that reflect true improvements in health outcomes. This article discusses the pitfalls of measuring the success of early detection tests for cancer, particularly in the context of screening programs, and provides illustrative examples that demonstrate how commonly used metrics can be deceptive. Early detection can result in downstaging (favourable stage shift) when more early-stage cancers are diagnosed, even without reducing late-stage disease, potentially leading to overdiagnosis and overtreatment. Survival statistics, primarily cancer-specific survival, can be misleading due to lead time, where early detection simply extends the known duration of the disease without prolonging actual lifespan or improving overall survival. Additionally, the misuse of relative measures, such as proportions, ratios, and percentages, often make it impossible to ascertain the true benefit of a procedure and can distort the impact of screening as they are influenced by diagnostic practices, misleadingly improving perceived mortality reductions. Understanding these biases is crucial for accurately assessing the effectiveness of cancer detection methods and ensuring appropriate patient care.

## 1. Introduction

Cancer remains one of the leading causes of mortality worldwide [1], making the early detection of malignancies a crucial component of preventive medicine. The premise is simple: identifying cancer at an earlier, more treatable stage should theoretically lead to better patient outcomes and reduced mortality [2]. This goal is typically pursued through screening—a type of medical examination or procedure carried out on people in a symptom-free group to determine their likelihood of having a certain disease [3]. 

However, while this approach holds promise, the metrics used to evaluate the success of early detection are complex and can be misleading. Currently, routine screening is recommended only for certain types of cancer (usually breast, cervical, colorectal, and lung cancer), but the majority of cancer deaths are caused by malignancies without recommended screenings [4]. This gap has driven research and development toward detecting a broader range of cancers through non-invasive methods, such as liquid biopsies, as well as through predictive, AI-powered algorithms based on imaging.

In recent years, there has been a growing interest in multi-cancer early detection (MCED) tests, which aim to identify multiple cancer types from a single sample, typically blood [5]. The potential of these tests has generated significant attention, with the market for MCED projected to reach USD 23.6 billion by 2031 [6]. As the enthusiasm for MCED tests increases, so too does the need to critically evaluate whether cancer screening, including these new technologies, genuinely saves lives. 

The question of the true efficacy of cancer screening becomes even more important in light of the ongoing large-scale evaluations for novel early detection tests [7]. As clinical trials progress, it is imperative to establish robust and meaningful criteria for success that accurately reflect tangible improvements in health outcomes, rather than relying solely on measures that do not fully capture the complexities of cancer progression and patient survival and can be misleadingly used to flatter the advantages of early detection. 

## 2. Increased Detection of Early-Stage Cancers

The NHS is currently conducting a £150 million industry-funded trial, involving over 100,000 people in England, to evaluate the effectiveness of a cell-free DNA-based MCED test [7]. The trial’s “success criteria” reportedly include achieving a 75% increase in cancer detection compared to a control group [8]. While such metrics may seem reasonable at a first glance, increased detection rates do not necessarily translate into better health outcomes for patients. The additional cancers identified may include indolent or slow-growing tumours that would not have caused harm during the patient’s lifetime—a phenomenon known as overdiagnosis [9]. This issue is best exemplified by thyroid and prostate cancers, where increased diagnostic scrutiny has led to a significant rise in detected cases, sparking debate over the potential for harm due to overdiagnosis [10,11].

For example, the incidence of thyroid cancer has surged in recent decades, largely driven by the detection of small, asymptomatic tumours through advanced imaging [12]. Many of these detected thyroid cancers are papillary microcarcinomas, which often have a very low risk of progression and may never cause symptoms or require treatment. Consequently, the increased diagnosis of these small tumours has not necessarily led to a corresponding reduction in mortality, raising concerns that patients may undergo unnecessary treatments that carry their own risks. The overdiagnosis of thyroid cancer has been estimated at 90% in South Korea; 70–80% in the United States, Italy, France, and Australia; and 50% in Japan, the Nordic countries, and England and Scotland [13], and has a significant impact on the total costs of thyroid cancer management [14].

Similarly, in prostate cancer, the widespread use of prostate-specific antigen (PSA) testing has led to a marked increase in the detection of prostate cancers, including many low-grade tumours that may never become life-threatening [15]. The challenge lies in distinguishing between aggressive cancers that require treatment and those that are unlikely to shorten a patient’s lifespan. Overdiagnosis in prostate cancer can lead to overtreatment, including surgeries and radiation therapy, which can cause significant side effects, such as incontinence and sexual dysfunction, without providing a clear survival benefit [16]. An increase in cancer detection without a corresponding reduction of late-stage disease and/or of cancer-related deaths has also been reported for breast [17], lung [18], and kidney [19] cancers and melanoma [20]. 

**Figure 1:**
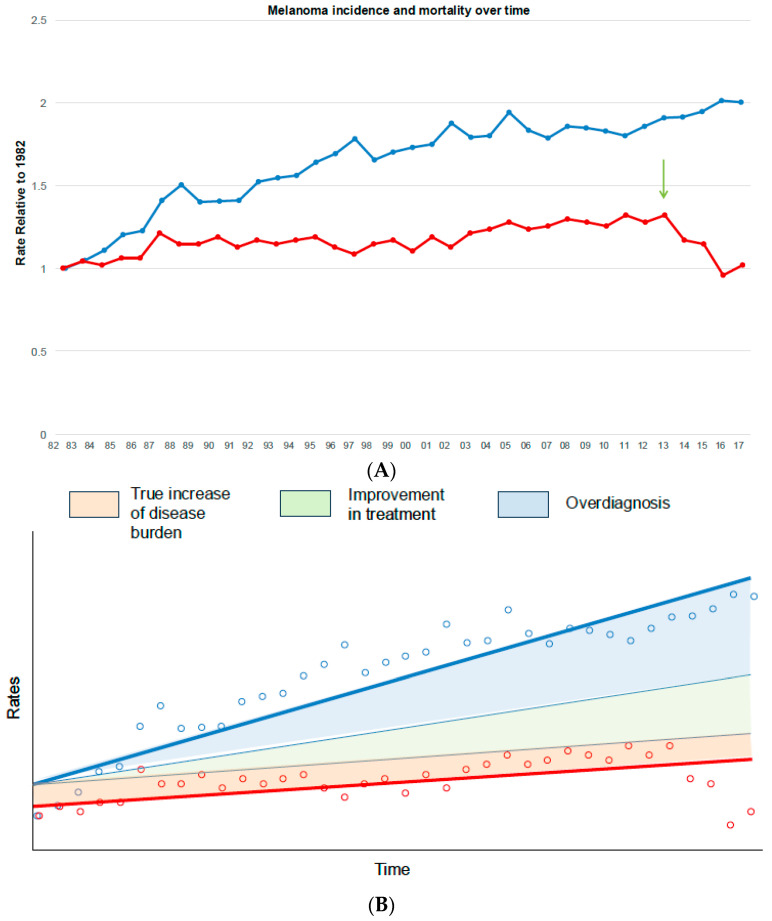
Melanoma incidence and mortality trends in Australia. (**A**) The graphic depicts the relative rates (incidence, blue line; mortality, red line) over time between 1982 and 2017, age-adjusted to the Australian population according to the original Cancer Australia registry [22]. The rates in 1982 serve as the reference group. The arrow depicts the introduction of checkpoint inhibitors in the treatment of melanoma. (**B**) The graphic schematically depicts the most common occurrences that could explain a gap between incidence and mortality rates over time, namely, the true increase in incidence, with or without improved treatment, and overdiagnosis. The tinier blue lines represent the incidence trends that we would expect to have compared to mortality (red line) if the gap was only explained by increased background incidence (incidence and mortality grow in parallel) and improved treatment (the disparity between incidence and mortality increases).

The epidemiological signature of overdiagnosis is an increase in the incidence of disease in spite of stable mortality [21], where the latter is viewed as a marker for stable true cancer occurrence. As an example, let us consider the incidence and mortality for melanoma in Australia using a national cancer registry [22]. Trends were illustrated using the relative age-adjusted rates over time, in which the 1982 rate serves as the baseline (Figure 1). Compared with the baseline (year 1982), incidence doubled, whereas mortality increased by approximately 30% over 30 years, with a peak in 2013. From 2014, there was a sharp reduction of deaths (−38% within 3 years), likely due to the efficacy of novel treatments such as checkpoint inhibitors, whereas the age-standardised incidence rate continued to rise (Figure 1). While it is conceivable that the observed mortality could be the result of a simultaneous rise in true cancer incidence and advancements in treatment over time, such a perfect annual balance of these opposing factors would be an extraordinary—and unlikely—coincidence [21]. In this specific example, there might have been a true increase in disease burden in Australia, since mortality rates also increased. Even assuming that improvements in medical care were responsible for reducing melanoma mortality by a certain extent [23], there would still be a large divergence between diagnoses and deaths (Figure 1B). Furthermore, when novel, effective treatments such as immunotherapy break though, this generally leads to a sharp decrease in case fatalities within a short timeframe. Accordingly, the introduction of an effective therapy in Australia could be inferred from mortality trends as a reduction of mortality (Figure 1A, arrow), likely due to a reduction in melanoma case fatalities, which can be observed post 2013. Thus, the trends shown in Figure 1 strongly suggest that overdiagnosis is responsible, at least in part, for the increase in melanoma diagnoses in Australia. 

The debate over overdiagnosis underscores the importance of carefully defining the success criteria in cancer screening trials. While detecting more cancers may appear beneficial on the surface, the ultimate goal should be to improve patient outcomes by focusing on clinically significant cancers that would benefit from early intervention. As a consequence, the primary outcome of screening or early detection tests should be the reduction in mortality rather than an increase in detection. 

## 3. Favourable Change in Stage Distribution (“Stage Shift”)

A change in the distribution of cancer stages, known as “downstaging”, is a common metric in cancer epidemiology and is often referred to as favourable “stage shift”. Early detection efforts aim for a favourable stage shift, marked by an increased proportion of early-stage cancers. Conversely, an unfavourable stage shift, characterized by a higher proportion of late-stage cancers, is often seen as evidence of late diagnosis due to reduced screening. 

However, these shifts in cancer stage distribution can be misleading and do not always equate to better outcomes for patients [24]. To illustrate this, let us consider the detection of early-stage cancers through an intensified screening program. While such a program may increase the number of cancers diagnosed at an early stage, this does not necessarily lead to a corresponding decrease in the incidence of late-stage cancers. In some cases, the total number of cancers detected might increase due to the identification of indolent tumours that would not have progressed to a more advanced stage or even become clinically relevant during the patient’s lifetime. This phenomenon can create the illusion of success—more early-stage cancers detected—without a true reduction in the burden of advanced, life-threatening disease.

For example, in breast cancer screening, there has been a significant increase in the detection of ductal carcinoma in situ (DCIS), a type of non-invasive breast cancer often found through mammography [25]. While DCIS is considered an early-stage cancer, it is not always clear whether all cases would progress to invasive cancer if left untreated. The detection and treatment of these cases contribute to a favourable stage shift, but the overall impact on breast cancer mortality remains a subject of debate, as the incidence of late-stage breast cancer has not declined proportionally in screened populations [26]. Therefore, the favourable stage shift observed due to screening is primarily the result of the additional detection of small/early tumours rather than the reduction of late-stage disease and ultimately of cancer deaths.

Understanding the pitfalls of stage distribution is of paramount importance for estimating the benefit of cancer screening, as stage shift is often considered a measure of success. This is because detecting the effect of early diagnosis on mortality in a population may not be realistically achievable within a short timeframe; therefore, in randomized clinical trials of cancer screening, the incidence of late-stage cancer is sometimes used as a surrogate end point for cancer-specific mortality. For some cancer types, such as lung cancer and possibly breast cancer, two recent meta-analyses agree that late-stage disease can serve as a reasonable proxy [27,28]. However, for other cancer types like prostate cancer, where the natural history of the disease is more variable and many detected cancers are indolent, late-stage cancer incidence may not adequately reflect the true impact on mortality [27]. While controversy does exist on this matter [28], it is clear that a favourable stage-shift expressed as proportions may not correlate with a meaningful reduction in deaths.

Ultimately, what we should aim to measure is the stage-specific incidence of cancer—how many cancers are detected at each stage, and whether the number of cancers diagnosed at a late stage is genuinely decreasing. An increase in early-stage disease paralleled by an absolute reduction in late-stage cancer incidence, combined with declining overall cancer mortality, would provide stronger evidence that a screening program is truly effective. Without this information, relying solely on stage shifts can lead to overestimating the benefits of early detection and underestimating the potential harms, such as overdiagnosis and overtreatment.

## 4. Longer Survival and Mortality Statistics

The ultimate goal in detecting cancer, whether early or at a later stage, is to prevent or delay death from the disease [29], ideally without compromising quality of life. Therefore, the primary outcome of interest for cancer detection tests is whether screening and/or early diagnosis avert death or prevent the reduction of life quantity and quality due to the disease.

### 4.1. Longer Survival as a Flawed Measure of Success

In evaluating the effectiveness of cancer screening, cancer-specific survival—the time from diagnosis until death from the specific cancer—usually in the form of progression-free survival, is often used as a measure of success [29]. However, this metric can be misleading due to a phenomenon known as lead time bias. Lead time is the period between the earlier detection of symptomless cancer (owing to screening or simply to increased scrutiny) and the point at which the cancer would normally be diagnosed based on symptoms [30]. Lead time bias occurs when screening detects cancer earlier than it would have been discovered without screening, leading to an apparent increase in survival time from the point of diagnosis. However, this extended survival time may not reflect an actual extension of the patient’s overall lifespan; rather, it may simply represent a longer period during which the patient knows about the disease without any real benefit in terms of longevity. This can give the false impression that early detection is more beneficial than it truly is.

Another bias affecting survival statistics in cancer screening is length bias [31]. Length bias occurs because screening is more likely to detect slow-growing, less aggressive cancers, which have a longer preclinical phase (the period when cancer is present but asymptomatic). This makes these cancers more likely to be found through screening. As a result, screening may appear to improve survival rates, but this could simply be because it detects cancers with better prognoses, regardless of the screening.

Overdiagnosis bias is another critical concern, alongside lead time bias and length bias, when evaluating cancer screening effectiveness [32]. Overdiagnosis occurs when screening detects cancers that would not have caused symptoms or death during a patient’s lifetime. This type of bias can inflate survival statistics without actually benefiting the patient’s overall health outcomes.

Moreover, patients with early-detected cancers may receive treatments that have their own risks and side effects, potentially leading to deaths from causes unrelated to the primary cancer, which is why overall survival, rather than cancer-specific survival, should be considered when estimating the benefits of population screening. For example, certain chemotherapy drugs, like anthracyclines (e.g., doxorubicin), are known to cause heart damage [33]. This can lead to heart failure or other cardiovascular events, which might result in death years after the cancer treatment is completed. Similarly, radiation and chemoradiation therapy in survivors for non-Hodgkin’s lymphoma, especially when used in younger patients, determine an increased risk of developing a second, different type of cancer later in life [34]. Overall survival refers to the time from diagnosis to death from any cause, and it encompasses not only deaths due to the specific cancer but also deaths from other causes. Because it uses death from all causes as the endpoint (as opposed to death from a specific cause, which can be misattributed), overall survival is the most reliable and available survival measure [35]. These examples underscore the need for caution when interpreting survival statistics as indicators of screening success.

Despite crude survival statistics being a flawed measure of success for early detection programmes, they continue to be used. For example, a recent analysis of the NHS plan to improve cancer survival rates highlighted concerns about the over-reliance on survival statistics [36]. The plan aims to increase the proportion of cancers detected at an early stage, which could lead to improvements in cancer-specific survival figures; specifically, “from 2028, 55,000 more people each year would survive their cancer for at least 5 years following diagnosis” [37]. Similarly, at a recent launch of an IARC Handbook [38], “an improvement in 5-year survival rates by early detection to save lives” was provided as an example of a success story for that WHO-backed cancer prevention programme. However, if these improvements are primarily due an increase in diagnoses and are skewed by lead time, length time, and overdiagnosis biases—where cancers are detected earlier but patients do not live longer overall—the perceived success may not translate into actual reductions in mortality.

### 4.2. Survival vs. Mortality

Another misleading concept that can result in deceptive messages to the public is the confusion, particularly in the mass media, between survival and mortality rates. While in everyday language, survival and mortality might seem like two sides of the same coin, in cancer statistics, they are fundamentally different measures [35]. Survival refers to the proportion of patients (i.e., people with cancer) who are still alive after a certain period following diagnosis, often expressed as a 5-year or 10-year survival rate (case-based measure). Mortality, on the other hand, is the rate at which people die from the disease within the entire population over a specified period of time (population-based measure). It is important to understand this distinction, as improvements in survival rates do not necessarily mean that fewer people are dying from the disease; it could simply reflect earlier detection without a true extension of life expectancy.

There is an ongoing debate about whether all-cause mortality, rather than cancer-specific mortality, should be the primary measure of success in cancer screening. Proponents of using all-cause mortality argue that it provides a more comprehensive picture of a screening program’s impact on patients’ lives, as it considers whether early detection truly extends life rather than merely prolonging the time people live with a diagnosis. It also considers the potentially negative impact of treatments and medications on patients’ health. Using all-cause mortality or life-years gained as a success measure, virtually no screening would be justified [39]. However, opponents question the suitability of all-cause mortality as a measure of success in cancer screening. One reason for this is that many cancers individually have a relatively low incidence in the population, meaning that changes in cancer-specific outcomes may not significantly affect overall mortality rates at the population level [40]. Additionally, focusing on deaths for all causes can obscure the benefits of early detection for certain cancers. This is because the potentially significant gains seen in a few individuals (cancer-specific deaths prevented by screening) are averaged across the entire screened population, diluting the apparent benefit [41].

The dichotomy between cancer-specific and all-cause mortality might be overcome when testing the efficacy of MCED, as recently pointed out [42]. All-cause mortality can be a valuable endpoint for MCED tests, particularly those capable of detecting a wide range of cancers, including those with high incidence rates such as lung and colon cancers. These types of tests have the potential to impact overall mortality significantly, given their broader scope and ability to identify cancers that contribute substantially to death rates. However, for MCED assays that are designed to detect only a limited number of cancer types, or that focus on cancers with relatively lower incidence, using all-cause mortality as an endpoint may not be appropriate or informative. In such cases, the tests’ impact on overall mortality might be less pronounced or more difficult to measure, as they might not capture a significant portion of the cancer burden that contributes to overall deaths. Hence, while it is not feasible to test all-cause mortality when screening for an individual cancer, it may be meaningful to test all-cause mortality for multicancer screening when cancer deaths are a large component of deaths in general.

In conclusion, while improved survival is a critical goal of cancer screening, interpreting survival statistics requires careful consideration of the biases and complexities involved. Both disease-specific and overall case-based (survival) and population-based (mortality) metrics have their advantages and limitations, and the choice of which to use as a measure of success should be guided by the specific context of the screening program and the characteristics of the cancer being detected. Ultimately, the most meaningful outcomes are those that reflect real improvements in both the length and quality of life for patients.

## 5. Favourable Relative Changes: Ratios, Proportions, and Percentages

A common issue underlying the biases in the metrics discussed so far in the context of early detection is the use of relative changes, particularly in the form of ratios, proportions, and percentages. Relative changes in risk, incidence, mortality, or survival can be deceptive when not accompanied by clear information on absolute changes. These relative metrics can give a misleading impression of the effectiveness of early detection or screening programs, especially when the absolute numbers involved are small.

For example, consider a scenario where a new screening test for a particular type of cancer reduces the relative risk of dying from that cancer by 50%. At first glance, this might seem like a highly significant achievement. However, if the absolute risk of dying from this cancer is very low to begin with—say, 2 in 10,000—the 50% reduction would mean that the risk has decreased to 1 in 10,000. While this is a measurable improvement, the absolute benefit to an individual is minimal, and the large percentage reduction may exaggerate the perceived impact of the screening. This is best exemplified by breast cancer screening—the epitome of a successful screening programme. For a 50-year-old woman, who is in the target/risk group due to age, the estimated risk of having a screen-detected breast cancer in the next 10 years is 1.9% and her 20-year risk of breast cancer death is ~1%. As mammography reduces this risk by ~20% at best, the risk of death in the absence of screening should be ~1.25% [43]. Hence, if risk reduction owing to breast cancer screening is 20% in terms of relative change, the same translates to only 0.25% (or 2–3 in 20,000 person/years) in terms of absolute change.

Relative changes can be further influenced by the incidence of the disease in the population. For instance, if a screening program detects more cases of cancer due to increased sensitivity, the incidence of the disease will rise due to the detection of early cancer—as we have seen above in regard to stage shift. This holds true when stage distribution is expressed as odds ratios (ORs). OR is a statistical measure used to compare the odds of an event occurring in one group to the odds of it occurring in another group. If stage distribution is expressed using ORs, a screening program that detects more early-stage cancers will show a favourable change in the OR comparing early versus late-stage detection between screened and unscreened populations. This misleading output is best exemplified by cancer statistics during the pandemic, when screenings were suspended. In the United States, this translated to lower odds of being diagnosed with stage I disease in 2020 than in 2019, and higher odds of being diagnosed with stage IV disease in 2020 compared to 2019 [44]. However, the incidence of late-stage disease remained stable during the pandemic. The observed unfavourable stage shift was therefore simply due to a decrease in early diagnoses (stage I disease) owing to the temporary interruption of screening programs. This can give the false impression that the distribution of disease stages has changed, which might be misleading if not properly contextualized.

Relative changes can also be deceptive when measuring the proportions of cancer patients who die relative to those diagnosed, a metric called the Mortality-to-Incidence ratio (MIR) or Incidence-to-Mortality ratio (IMR). The MIR/IMR provide an estimate of the proportion of diagnosed patients who die from the disease, giving insight into its lethality, and is regarded by some as a marker that reflects the efficacy and availability of screening interventions and treatment outcomes and that can be used to influence public health strategy [45]. However, this metric can once again be affected by changes in diagnostic practices and hence incidence rates. For example, a rise in incidence owing to screening can lead to a higher IMR (or lower MIR). This could give the false impression that the disease has become less deadly or that the outcomes of treatment have improved, when in fact it may just be the result of detecting more cases that were previously undiagnosed. For example, the IMR for melanoma in the US increased from 3.9 to 14.0 (+261.4%) from 1975 to 2010, suggesting that the disease had become less deadly. However, this disproportionate temporal increase in the IMR was driven by a comparatively greater rise in melanoma incidence, while mortality remained stable [46]. Consequently, a higher IMR or lower MIR following the introduction of a screening program might be misinterpreted as a success in reducing mortality, even if the actual number of deaths has not changed.

The issue with the use of relative measures is particularly pertinent in public health messaging, where favourable relative changes are often highlighted to promote the effectiveness of screening programs. Without accompanying absolute figures, patients and healthcare providers may overestimate the benefits of screening, leading to an increased uptake of tests that may offer limited real-world benefit or even contribute to overdiagnosis and overtreatment. For example, women who were provided with detailed information were less likely to express an intention to undergo mammography [47]. Therefore, when evaluating the effectiveness of early detection methods, it is crucial to consider both relative and absolute changes to communicate the outcomes transparently. This dual approach provides a more accurate and balanced understanding of the true impact of screening programs on patient outcomes, ensuring that decisions are made based on meaningful improvements in health rather than potentially misleading statistical representations.

## 6. Concluding Remarks

The landscape of cancer screening and early detection is fraught with complexities and challenges that necessitate a nuanced approach to evaluation. While the premise of identifying cancer early to improve patient outcomes and reduce mortality remains compelling, this simple logic can be deceptive. The metrics used to gauge success in screening programs must evolve to reflect the true health benefits accurately (Table 1).

Firstly, the emphasis on increasing the detection of early-stage cancers (e.g., through MCED tests) illustrates a growing trend towards broadening screening capabilities beyond the traditionally recommended cancers. However, as demonstrated in ongoing trials such as the NHS-funded initiative, achieving higher detection rates does not automatically translate into improved patient outcomes. The phenomenon of overdiagnosis, particularly evident in thyroid and prostate cancers, underscores the potential harm of detecting cancers that may never progress to clinically significant stages.

Furthermore, the concept of stage shift in cancer epidemiology highlights another aspect of screening evaluation. While detecting more cancers at an early stage is desirable, it does not necessarily correlate with a reduction in late-stage disease or cancer-related mortality. This discrepancy challenges the simplistic interpretation of early detection as a panacea for improving survival rates.

Survival statistics, both cancer-specific and overall, play a crucial role in assessing the impact of screening programs. Yet, they are fraught with biases such as lead time bias, which can exaggerate the perceived benefits of early detection without necessarily extending patients’ actual lifespans. The debate over which survival and mortality metric—cancer-specific or overall—is most appropriate further complicates the interpretation of screening efficacy, especially given the varied natural histories of different cancers.

Relative changes in metrics like risk, incidence, mortality, or survival also pose challenges in the evaluation of screening effectiveness. Highlighting relative improvements without considering absolute changes can lead to misconceptions about the true impact of screening on public health outcomes, potentially fostering unrealistic expectations among patients and healthcare providers alike.

In conclusion, advancing the field of cancer screening requires a shift towards more unbiased and patient-centred evaluation metrics. Success should be measured not solely by increased cancer detection rates or stage shifts but by meaningful reductions in cancer mortality and improvements in quality of life. As we navigate the complexities of screening efficacy, it is imperative to select appropriate measures of success that prioritize the health and well-being of individuals.

## Figures and Tables

**Table 1:** Summary of commonly adopted measures of success of early detection and possible flaws.

Measure	Deceptive Reason for Success	Alternative Interpretation
Increased detection of early cancers	Identifying cancers at a more treatable stage will lead to better patient outcomes and reduced mortality	Incidence increases due to the detection of small, asymptomatic tumours through advanced technologies
Stage distribution	A favourable stage shift is marked by an increased proportion of early-stage cancers compared to late-stage	A favourable stage shift may result from more early-stage cancers detected without a true reduction in the burden of advanced disease
Survival	Early detection and/or early-stage diagnosis leads to an increase in the survival of cancer patients	Early detection introduces lead time, leading to an apparent increase in survival time from the point of diagnosis, without an actual extension of the patient’s overall lifespan
Relative changes	A reduction in the risk, case fatality, or increase in survival of the screened compared to non-screened group (e.g., a 20% decrease of case fatality rate (CFR) means that the cancer is less deadly)	Ratios, proportions, and percentages are influenced by both terms (e.g., CFR is influenced by incidence and hence by diagnostic practices) and can be deceptive when not accompanied by clear information on absolute changes

## Data Availability

Data are available upon reasonable request to the author. Data mining was undertaken on publicly available or previously published data.
